# Prostate sparing radical cystectomy and neobladder: institutional long-standing qualified consideration

**DOI:** 10.1186/s43046-025-00316-9

**Published:** 2025-09-08

**Authors:** Omaya Nassar, Iman Gouda Farahat

**Affiliations:** National Cancer Institute of Cairo University, Giza, Egypt

**Keywords:** Prostate sparing cystectomy, W-neobladder, Y-neobladder, Ileocecal neobladder, Orthotopic bladder continence, Neobladder morbidity, Urodynamic of bladder substitutions

## Abstract

**Objectives:**

To balance the extended functional urinary voiding and morbidity outcomes amid Ileal W and Y-shaped contrasted to spherical ileocoecal (IC) orthotopic bladders subsequent prostate-sparing radical cystectomy (PRC) versus standard radical cystoprostatectomy (RC).

**Material and methods:**

Two hundred eight male bladder cancer patients were grouped into 98 RC followed by 43-W, 31-Y, and 23-IC in comparison to 110 PRC followed by 35-W, 37-Y, and 38-IC. The functional voiding outcomes were determined by detailed patients’ interview and urodynamic studies (UDS).

**Results:**

Twenty-four hour pad-free continence for PRC at the 6th month was (37, 25, and 36%) as regards W, Y, and IC in that order, improved to (51, 44, and 51%) at the 12th month and (68, 70, 73%) at the 5th year. Nocturnal continence at the 12th month was (60, 56 and 59%). After 73 months median surveillance, day-time control was 90% equal for the 3 pouch configurations. RC patients faced inferior continence rates exclusively during nocturnal time. Urethral pressures started higher for PRC and increased over time, resulting in diminution of stress frequency.

Delayed pouch morbidities were 41% for W vs. 22.4% and 25% among Y and IC series. Urethral recurrences were non-existent in both groups without atypical or metaplastic changes. Mucosal pattern persisted in 60% while muscular coat atrophied in 83% of ileal and 40% of colon walls.

**Conclusion:**

Voiding control parameters for PRC were significantly superior. Though early continence is in favor of W pouches, delayed observation showed equivalent Y and IC results besides least delayed pouch-related morbidities for IC.

## Introduction

Radical cystectomy (RC) diverted by orthotopic neobladder is a major intricate surgical technique with variable and gradual voiding control. Continence is the result of interaction between outlet resistance and intraluminal pressure. Intrinsic urethral tension is governed by smooth and striated muscular activity, and its proximal part is exposed to the transmitted intra-abdominal pressure [1–3]. Neoadjuvant chemoradiation increased the scope of conservative resection in muscle invasive bladder cancer, increasing suggestions of PRC without increased risk of urethral recurrence [4, 5].

Regardless of the type of urinary diversion, the procedure should not compromise cancer control and should have an acceptable delayed complication and reoperation rates [1, 6–8].

An effective orthotopic neobladder should have some of the several characteristics of normal bladder, viz. a continent mechanism, adequate capacity with compliance, and an antireflux mechanism preventing upper back pressure [2, 3, 6–9].

Detubularized W-shaped double loop and Y-shaped ileal pouch offer large capacities immediately postoperative [6–11].

IC with adequate compliance and capacity has a low incidence of electrolyte changes, over distension, and residual urine. IC is particularly well suited for orthotopic diversion because of its consistent vessels, simple construction, and easy descent into the pelvis [12, 13].

This prospective comparative study preserves proximal urethral length together with the bladder neck sphincter to improve continence, both subjective and objective. Proximal urethral sparing results parallel the outcomes of 3 pouches W, Y, and IC [14, 15].

## Patients and methods

In 1999, an institutional review board trial was conducted to recruit male patients with T_1-3_ bladder cancer with or without neoadjuvant chemotherapy and/or EBRT. Enrollment criteria were (1) tumors 2 cm away from the bladder neck (dome or posterior wall) without in situ. (2) Adequate preoperative continence without urethral stricture. (3) Creatinine ≤ 150 µmol/L and normal liver function (4). Cooperative mental and intellectual capability to follow instructions, beside a written informed consent.

Withdrawal criteria included: 1—diabetes or intestinal diseases, 2—females. 3—solitary kidney or upper urological disease. 4—BMI ≥ 30. 5—liver cirrhosis. 6—raised PSA. 7—Karnofsky scale ≤ 70%. 8—impaired cardiorespiratory functions.

Two hundred eight candidates were sampled with 95% ± 6.8% confidence level and randomized to two series, each around 50%. One arm underwent nerve-sparing radical cystoprostatectomy together with orthotopic reconstruction using either ileal W, Y, or ileocecal pouches. The other arm underwent nerve- and prostate-sparing radical cystectomy with similar three-pouch configurations. Enrolled patients were males of matching age groups, tumor pathology, neoadjuvant preoperative treatments, and renal function.

By 2018, encompassed candidates were 98 underwent RC (W *n* = 43, Y *n* = 31 and IC *n* = 23) and 110 underwent PRC (W *n* = 35, Y *n* = 37, and IC *n* = 38) (Table 1).

**Table 1:** Candidates’ characteristics and post-surgical complications

Clinical and pathological features	Early complications (90 days)	W (78) No (%)	CDC	Y (69) No (%)	CDC	IC (61) No (%)	CDC
Age 53(30–75) Tumor type Non-muscle invasive TCC 51 Muscle invasive TCC 108 Squamous Ca 45 Adeno Ca 4 Pre-operative treatment -Neoadjuvant chemo (3/4 cisplatin-based) 76 -Pelvic radiotherapy 36 - Intravesical BCG 23 Renal US and CT -Dilatation- 24 - Nephropathy 6	-Wound infection	8 (10.3)	I–II	9 (13)	I**–**II	7(11.5)	I**–**II
	-Abdominal dehiscence	3 (3.8)	III	2 (3)	III	4(6.5)	III
	-DVT	2 (2.6)	II	–		–	
	-Urine leakage ≥ 500 ml/24 h	16 (21)	II	5 (7.2)	II	5 (8.2)	II
	-Prolonged ileus	16 (21)	II	7 (10)	II–III	7 (11.5)	II**–**III
	-Intestinal leak	–	–	2 (3)	III, IV	3 (5)	III, IV
	-stress gastric ulceration	4 (5.1)	II	–		–	
	Total cases*	16 (21)		12 (17.4)		12 (19.7)	
	Reoperation	3 (3.8)		4 (5.8)		4 (6.5)	
	Mortality	–	–	2 (3)	V	2 (3.3)	V
	Delayed complications	W (No = 78)		Y (No = 67)		IC (No = 59)	
	-Uretero-enteric stricture	8 (10.3)	III	11 (16.4)	III	5 (8.5)	III
	-Repeated retention (bladder neck stricture) - Hypercontinence	12 (15.8)–	III	8 (12)–	III	4(6.8)–	III
	Upper infection	9(11.5)	II–III	6(9)	II–III	7(11.90	II–III
	- Acidosis - Mild and temporary - Severe	31 (39.7) 4 (5.2)	I II, IV	17 (25.3) 1 (1.5)	I II	7(11.9) 4(6.8)	I II, IV
Recurrence, mortality, and disease-free periods - Tumor cumulative relapse 43 (21%) 18–65 months - Tumor cumulative mortality 63 (18.3%) 27–78 months - Non-specific cumulative mortality 20(9.6%)							
	-Stone–pouch Renal	3 (3.8)	II, III	2 (3.2) 2 (3.2)	III III	1	II
	-Over distension -Spontaneous rupture	23 (29.5) 3 (3.8)	I and III III, IV, V	16 (24) –	I and III –	3(5.1) –	III
	-Prolonged diarrhea -Vit. D, B12 debit	**5 (6)**	**I**	**6 (9)**	**I**	**7 (11.5)**	**I**
	Total cases*	32 (41)		15 (22.4)		15(25.4)	
	Reoperation	6 (7.7)	V	9 (13.4)		3(5)	
	Mortality -Diversion specific -Tumor specific - Non-specific	3 (3.8) 16 (20.5) 3 (3.8)		2 (3.2) 11 (16.4) 2 (3.2)	V	– 16 (27) 4 (6.8)	

^*^More than one complication in a single candidate

CDC grades [16]: *I—*complication requiring allowed therapeutic regimens without surgical, endoscopic, or radiological interventions, *II—*more therapeutic regimens ± blood transfusions ± parenteral nutrition, *III—*required surgical, endoscopic, or radiological interventions, *IV—*life-threatening complication requiring IC/ICU management, *V—*death

### Contemplated technical phases

**Fig. 1:**
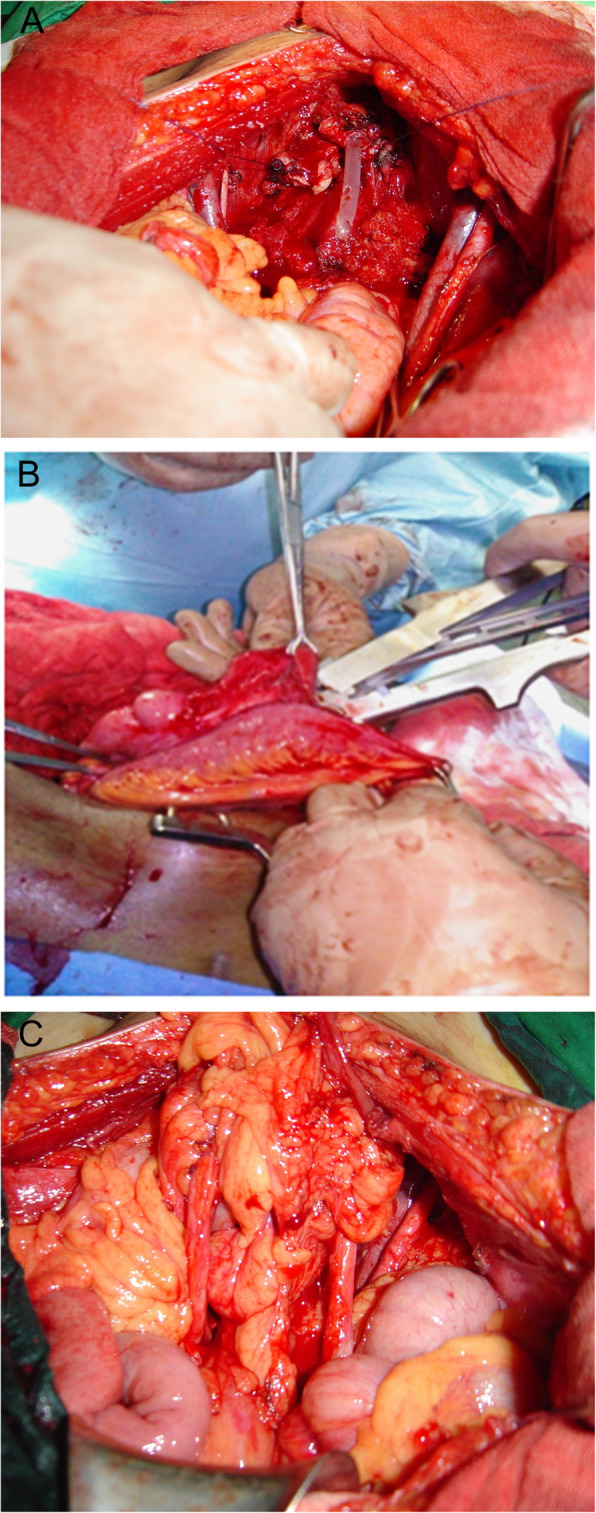
**A** The prostatic urethral stump after radical cystectomy. **B** Isolated pedicled ileocecal segment is stapled entirely side to side to create a spherical detubularized pouch. **C** Distal part of the pouch is implanted to the prostatic urethral stump using sutures (interrupted or continuous) and spatulated ureters are implanted directly end to side over stents (interrupted). Next to pouch closure, anterior wall is hung to the rectus muscle to avoid posterior sagging and later on hypercontinence

**Fig. 2:**
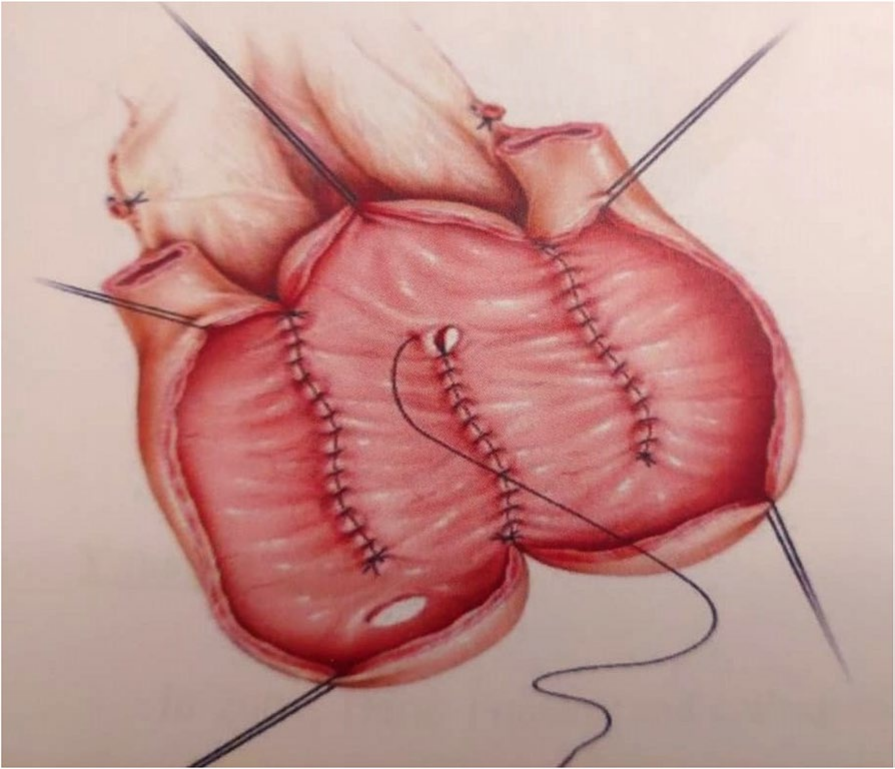
A diagram of 70 cm ileal segment configured in W and stapled parting proximal and distal 5 cm ends for end ureteric implantations

**Fig. 3:**
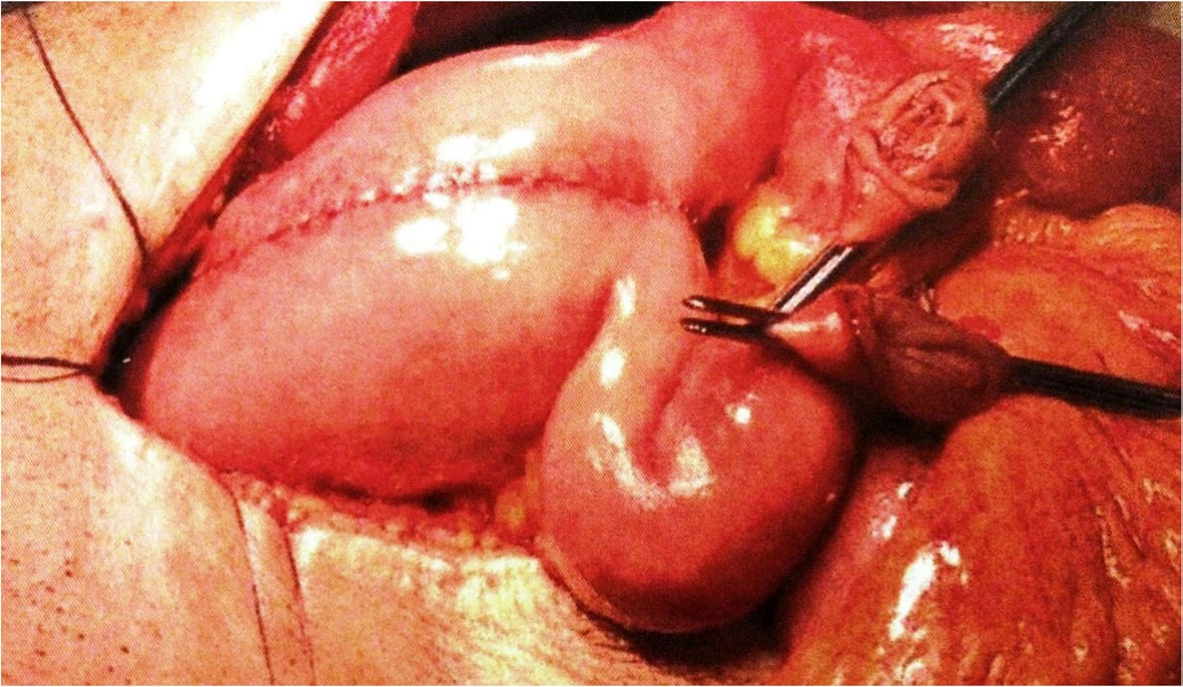
40 cm ileum is isolated in a Y configuration. Vertical limb was stapled side to side. Distal end was anastomosed to urethra and arranging for end-toto-end ureteric implantation

- Total or partial prostate saving was tailored by its US weight plus the frozen section [14, 15].
- Figs. 1, 2, and 3 illustrate configurations.
- Pouch was anastomosed to the urethra over a stent beforehand ureteric implantations and the constructed pouch was then anchored to the anterior abdominal wall together with posterior omentoplasty.
- Triluminal indwelling catheters were retained for an average 10 days and ureteric stents for 7 to 10 days, controlled by ascending pouchography.
- Patients were further advised to have liberal fluids till 4 h before sleep time. Candidates squatted to void every 2–4 h by Valsalva’s maneuver and were awakened to void every 4 h.
- Pelvic training, alkalinizers, urine antiseptics, and training for self-catheterization were instructed for sent home patients.
- Post-surgery adjuvant EBRT (30–35 Gy/5w) was designated for 122 patients with nodal involvement and/or deep muscular and perivesical fat infiltration.

### Surveillance

**Fig. 4:**
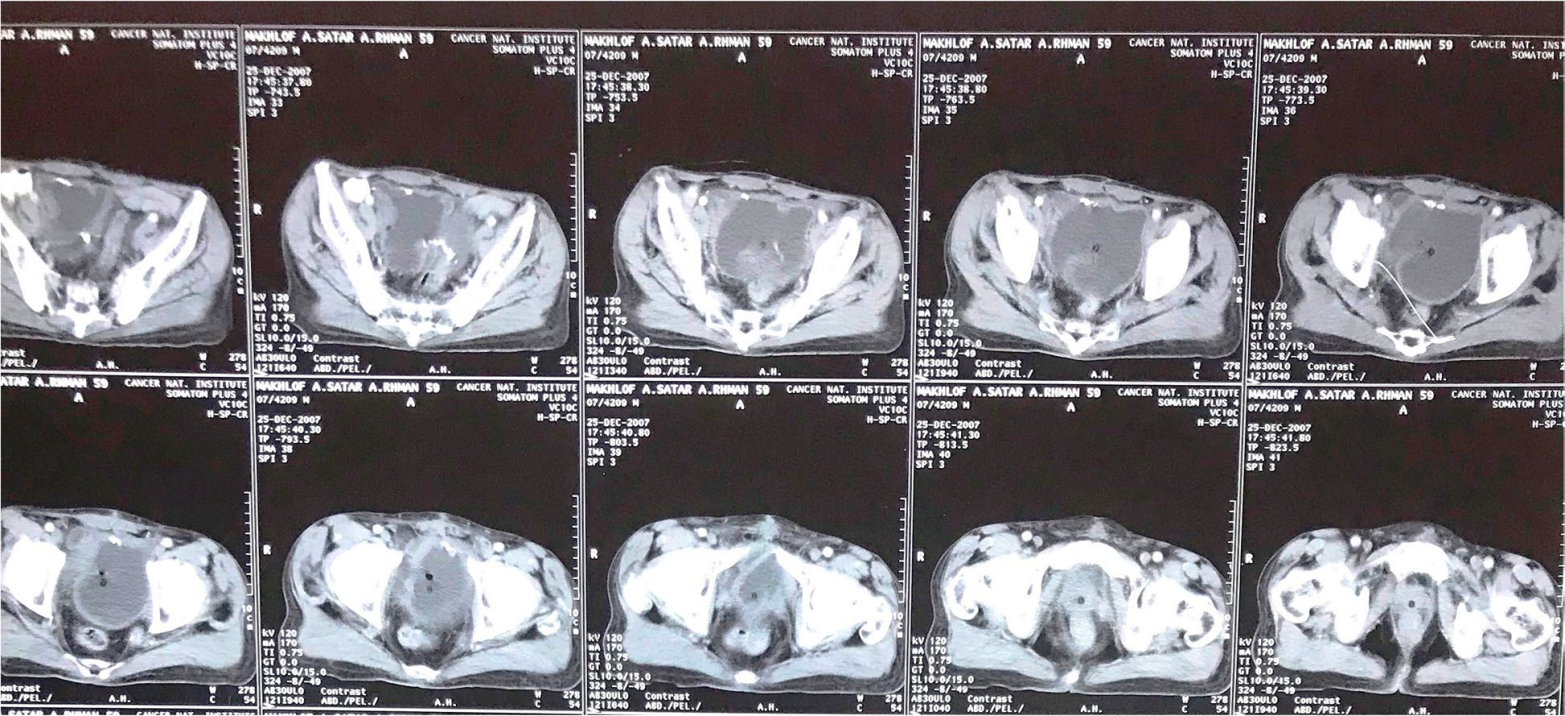
2-year postoperative CT shows post-evacuation cuts of IC neobladder after PRC. Pouch has 485 ml capacity. Anterior and posterior staple remnants are present without stones or encrustations on them

**Fig. 5:**
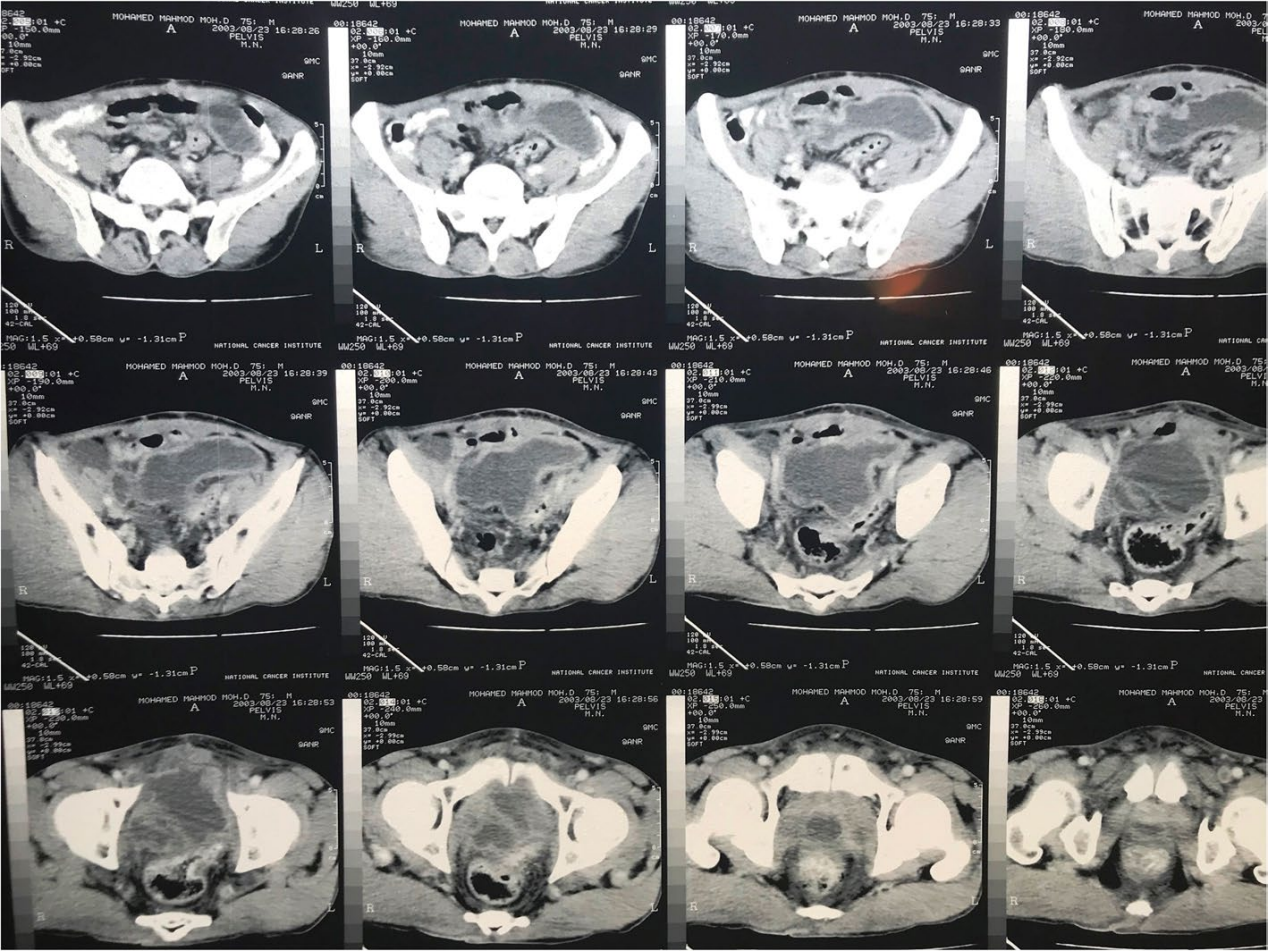
Post-evacuation CT cuts, 23 months post-RC and W diversion. There is pouch over distension and large 250 ml residual

**Fig. 6:**
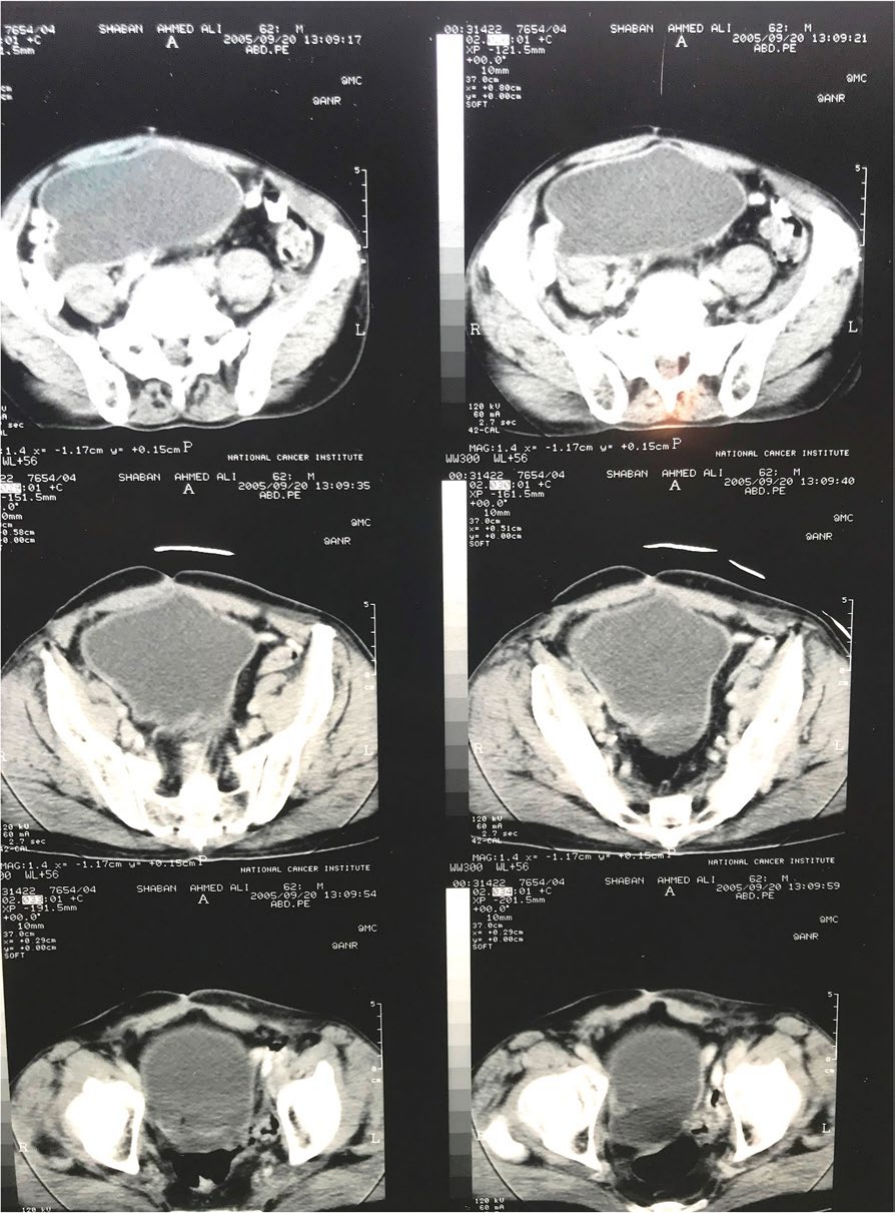
Ileal Y-shaped pouch post-PRC with 510 ml capacity and 200 ml residual

Scheduled observations/2-month evaluated the upper and lower urinary functional sequences besides oncologic results. Electrolytes and renal functions plus regular US, IVU, and CT were scheduled besides cystoscopy and interventional ureteric utilities whenever needed (Figs. 4, 5, and 6).

Continence was monitored commencing with the 2nd month evaluation by detailed patient voiding history. Pad-free between voids even with stress drips or urgency reflected as real control (Table 2). Scheduled urodynamic evaluation at the 6th and 12th months to bypass the maximum morbidity time and adjuvant irradiation effects (Table 3).

**Table 2:** Sequences of pad-free continent percent for proximal urethral sparing and classical cystectomy

	W				Y				IC			
	Day	Night	/24 h	Stress grades	Day	Night	/24 h	Stress grades	Day	Night	/24 h	Stress grades
2nd month	(53)	(13)	(–)	I (19), II (22), III (44)	(49)	(–)	(–)	I (49), II (31), III (15)	(41)	(8.2)	(–)	I (19), II (22), III(43)
6th month												
PSRC	(65)	(45)	(37.5)	I (32.5), II (25), III (7.5)	(70)	(25)	(25)	I(25), II (12.5), III (0)	(64)	(45.8)	(35.6)	I (26), II (13), III (2.5)
RC	(58)	(35)	(27)	I (41), II (26), III (12)	(61)	(16)	(15)	I (52), II (19), III (8)	(59)	(40)	(35)	I (41), II (26), III (12)
12th month												
PSRC	(86)	(60)	(51)	I (32.5), II (25) III (5)	(86)	(56)	(44)	I (42), II (5), III(–)	(88)	(59)	(51)	I (26), II (10), III (–)
RC	(66)	(50)	(40)	I (45), II (19), III (12)	(77)	(55)	(55)	I (32), II (13), III (–)	(75)	(50)	(50)	I (45), II (19), III (12)
5th year												
PSRC	(87)	(70)	(68)	I (16), II (3), III (–)	(85)	(70)	(70)	I(20), II (4), III (–)	(89)	(84)	(73)	I (8), II (–), III (–)
RC	(73)	(50)	(50)	I (39), II (10), III (–)	(76)	(47)	(47)	I (22), II (4), III (–)	(73)	(67)	(67)	I (39), II (10), III (–)
Time period to true day-time continence Total series: PRC 6(1–14) RC 11(1–36) *p* = 0.0150^(s)^ W series: PRC 3.5(1–11) RC 8 (1–22) *p* = 0.001^(s)^ Y series: PRC 4 (2–14) RC 11 (2–36) *p* = 0.0051^(s)^ IC series: PRC 6 (1–12) RC 12(5–22) *p* = 0.015^(s)^					Time period to true night continence Total series: PRC 12 (1–22) RC 18(2–39) *p* = 0.065 W series: PRC 6 (2–18) RC 11(6–22) *p* = 0.012^(s)^ Y series: PRC 7 (1–22) RC 7 (3–25) *p* = 0.15 IC series: PRC 3 (1–16) RC 12(2–39) *p* = 0.001^(s)^							

Number of continent candidates (pad free ± stress drips)

Urgency and stress incontinence grades: *I*—urine loss during coughing, sneezing, and pressure, *II*–urine loss during lifting, running, and climbing stair, and *III*–urine loss during standing without physical activity

**Table 3:** Urodynamic^#^ results

	6^th^ month			12^th^ month			
	W-	Y-	IC	W-	Y-	IC	
	(Nƍ* = 37)	(Nƍ = 42)	(Nƍ = 48)	(Nƍ = 30)	(Nƍ = 28)	(Nƍ = 31)	
**Cystometry**							
-Capacity(ml)	510(360–660)	433(320–601)	413(335–590)	514(360–690)	502(410–656)	533(362–626)	0.056
-Residual urine (ml)	78.3(20–200)	25(0–80)	36(0–285)	102(20–350)	95(22–302)	45(0–347)	0.012^**S**^
-Basal pressure (cm H_2_O)	22.1(10–65)	25.5(21–70)	18 (9.1–63.5)	11(0–25)	17(10–78)	16(9–24.3)	0.112
-Maximal pressure (cm H_2_O)	26.3(15–45)	21.8(10.6–68)	39(14–85)	38.4(6–65)	35(16–102)	42 (19–98)	0.021 ^**S**^
**-Uroflowmetry**							
-Max. voiding volume (ml)	270(150–400)	220(142–367)	210 (120–388)	510(340–660)	398(351–701)	346(255–642)	0.012^**S**^
-Voiding time (s)	50(33–76)	44.5(32–66)	30 (21–55)	54.6 (20–135)	39(25–104)	35(22–90)	0.011 ^**S**^
***- RC urethral pressure*** (cm H_2_O) • Bladder neck • Voluntary urethral ***- PRC urethral pressure*** (cm H_2_O) • Bladder neck Voluntary urethral		6-month 22.5(9–30) 66(51–119) 36(17–49) ^**S**^ 0.0011 89(43–122) ^**S**^ 0.0213		12-month 20.4(15.5–36) 71(30–123) 37.6(11–51) ^**S**^ 0.041 102(39–119) ^**S**^ 0.0012			

^#^ UDS-600 software and standard water-filled external transducer-type device

^*^(Nƍ) candidates underwent study

^S^Significant

### Statistical analysis

*Data analysis applied SPSSwin statistical package version 23 (IBM Corp., Armonk, NY).* Numerical data is computed as mean ± standard deviation (SD), median (minimum–maximum) and qualitative as frequency (%). Univariate analysis applied for the significant factors affecting time to complete continence and urodynamic factors. Survival analysis used the Kaplan–Meier method. *A p-*value of ≤ 0.05 was considered significant.

## Results

### Voiding control

**Fig. 7:**
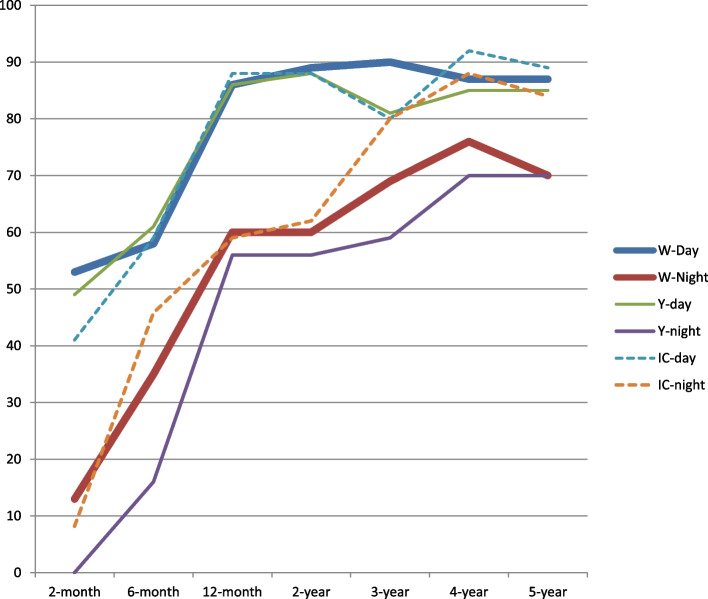
Day and night pad-free continence progress for the entire W, Y, IC pouches**.** Second month post-surgery; nearly 50% of the total study group was day continent, enhanced to 60 and subsequently 86% at the 6th to 12th months. Meanwhile, 2nd month nocturnal controls were relatively poor amid the three groups. Similarly, 2nd month stress incontinence was high in all groups; nonetheless, most stresses were grades I and II (79% for W patients in comparison to 95% for Y patients and 85% for IC). Sixth month night time continence improved significantly for the IC (46%) and W (45%) and to a less degree for Y (25%). After the 12th month day, night and total 24-h control improved much and was analogous for the 3 groups. Fifth-year observations described consistent improvement in night and total control for the 3 groups; moreover, superior for the IC group than W&Y groups (84 vs. 70% respectively, 0.0021)

Starting with the 6th month post-diversion, the PRC group had significantly enhanced voiding control over the RC group for both day and night times, nearly of equal rates among the three pouch varieties, and the median times to achieve real urine continence, either day or night, were all significantly shorter among PRC than that of the RC (Fig. 7).

Grades I and II stress drips persisted higher for W and IC and nearly doubled that of Y even though urethral pressures were similar. Average bladder neck pressures were [29.5 vs. 28.4 cm H_2_O for W vs. Y and IC *p* = 0.215]. Average voluntary urethral pressures were [98 vs.102 cm H_2_O for W vs. Y and IC *p* = 0.41]. PRC enhanced stress drip control significantly for the three pouch diversions, particularly in the IC group (Table 2).

**Fig. 8:**
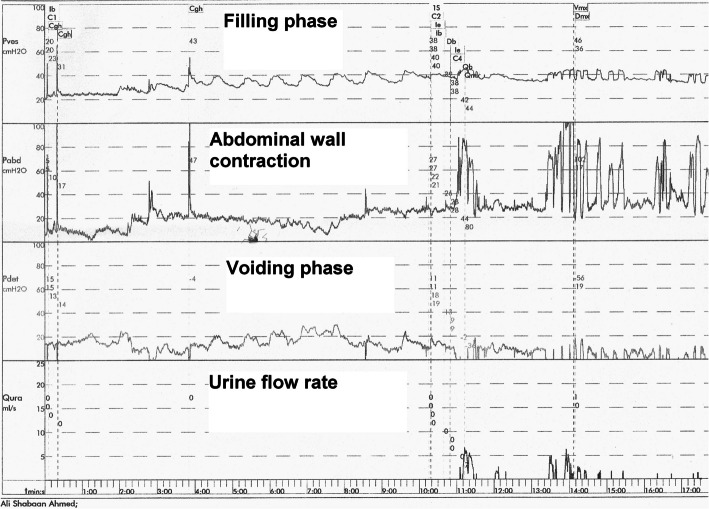
UDS 39-year-old male after PRC and IC (12th month) delayed sensation, 481 ml pouch capacity, no contraction during filling phase or during voiding. Patient voids by abdominal contraction (6.7 ml/s max flow rate), 3.2 ml/s average rate, and 210 ml post-voiding residual urine

Sixth month average capacities were inferior for IC and Y groups in contrast to W, but by the 1 st year later, there were no differences, and basal bladder pressures were similar (Table 3). Maximal pressure was significantly higher for IC in contrast to both W and Y. Twelfth month maximal voiding volume was significantly larger for W in contrast to Y and IC, indicating more pouch dilatation; similarly, voiding time was shorter for both IC and Y (Fig. 8).

### Morbidity and surveillance

Cystectomy and diversion time averaged 185 min (147–380) equal for PRC and RC. Average operative time was a little shorter for IC and Y-pouch than W (180 vs. 220). Mean blood loss was 550 ± 460 ml with 2 (0–5) transfused units without differences between PRC vs. RC or the pouch configuration. In-patient stays averaged 12 days in non-morbid and15 for all (10–49).

#### Early

Urine leaks were significantly less in PRC (9%) compared to 16% for the RC, but nearly equivalent regarding the detubularization technique (staples vs. sutures) (Table 1).

#### Delayed morbidity

The median surveillance time was 73 months (16–183) and late morbidities along 5-year observation were significantly higher amid the W group compared to the other groups (41% versus 22% (Y) and 25% (IC) 0.0012). Pouch-related drawbacks like metabolic acidosis, repeated retentions, and overdistension even with spontaneous delayed rupture were greater for the W group. Overdistension was noticed to start as early as 6th month for W patients and later after 2nd year for Y and IC, monitored by US and CT more frequently with repeated retentions and increased residuals. Three patients (> 18 months) passed because of pouch rupture.

Occurrence of benign bladder neck strictures coupled with repeated retentions was parallel among PRC and RC. Prostate dimensions in a few PRC patients older than 60th increased without notable PSA changes.

### Over 5-year surveillances

Patients’ satisfaction rates and pad free rates were parallel with the drop of grade I stress to 5% for both PRC and RC. Residual urine > 150 ml was persistent for 30% of W group (PRC and RC), 20% of Y cases, and 8% for IC. No newly developed uretero-enteric strictures were observed after 5th year, and renal functions and electrolytes were stable. Age-related changes in serum creatinine clearance were noticed.

Nearly equivalent for the 3 pouch groups’ serial liver enzymes spotted collectively 26 patients with raised AST and ALT who further (24–48 months) developed HCV cirrhotic changes in 13 candidates.

### Pouch wall alters

**Fig. 9:**
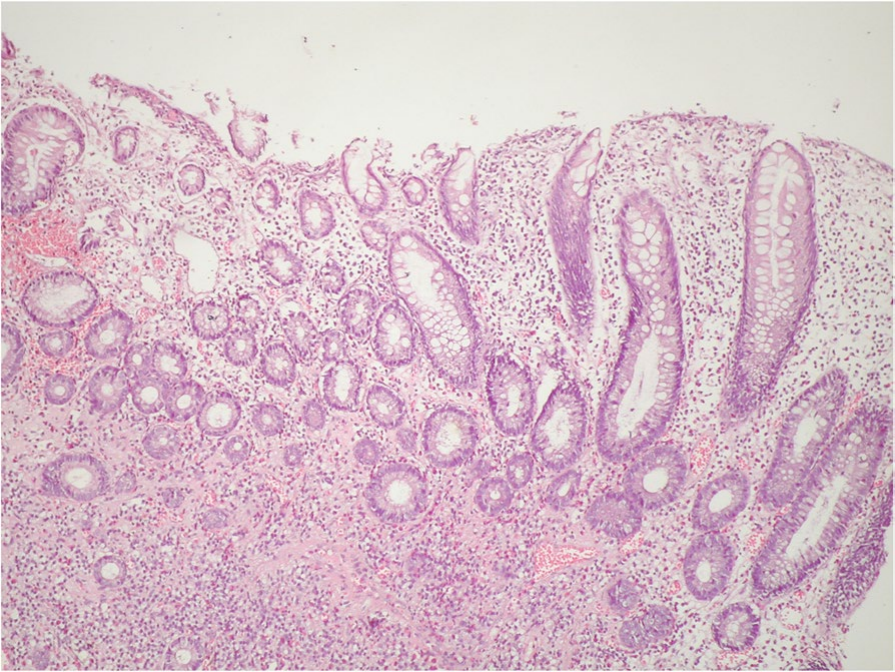
Pouch histopathology after 7 years. Persistent mucosal patterns without atypia, metaplasia, or dysplasia. Excess lymphocytic infiltration without metaplasia. Superficial muscular layer atrophic fibrosis

Pouch wall cold-cup biopsies in 30 patients of both ileal (*n* = 18) and colonic (*n* = 12) reserved their histologic mucosal pattern up to 11 years in 18 (60%) and atrophied in the others with neither metaplastic nor malignant changes (Fig. 9). Muscular layers atrophied in most of the pouch biopsies and swapped to thin fibrous layers with lymphocytic infiltration. Muscular layer atrophy for the ileal pouches was observed in 15/18 (83%) more than colonic walls 5/12 (41%).

### Oncologic aspect

Disease-free 5-year survival estimated 76% and 26 months median recurrence time. Local recurrence involved lateral pelvic wall with neither central nor urethral relapses. Local recurrences coupled with distant metastases in 22 patients. Recurrences were less frequent in patients who had EBRT (6.6% versus 16%). Survival and relapses were equally distributed among the two cystectomy patterns and the three pouch configurations. Local relapses strongly correlated with tumor histopathology and postoperative radiotherapy treatment.

## Discussion

Aim of bladder substitutes is the re-establishment of an almost normal physiological function of the lower urinary tract with the least drawbacks. A mean voiding volume of 350 ml, a maximal voiding volume of 500 ml, and frequency of micturition of 3 to 5 times during the day and once or twice at night with separating dryness may be regarded as physiological [1–3, 7, 17, 18].

Continence depends on adequate capacity besides intact urethral and pelvic floor sphincters, which are able to maintain resistance pressure across the urethral continence zone that exceeds the pressure generated within the pouch. Additional factors include urethral length and sensitivity, patient’s age and his mental status, intact pelvic nerve supply to the rhabdosphincter, completeness of voiding, and the presence or absence of bacteriuria [1–3, 7, 8, 14, 15].

Early assessment of valid functional results of continent diversions within the immediate few months is often biased by the healing of the multiple suture lines and anastomoses, the adjuvant radiotherapy and its linked morbidities. Furthermore, the psychosocial consequences of this reconstructive surgery and the ensued disturbed anatomy and physiology on the patient’s accommodation are significant. Pad-free control in between voids was deliberated as the true continence even with concomitant stress leaks. However, concerning patients’ satisfaction rates of continence, it was higher than pad-free rates. Most patients reflected using night condoms and 1 to 2 pads per day as a satisfactory control.

Many patients had gradual sensations of fullness simulating those of the normal bladder. This sensation is inherited from the already present bowel wall receptors and transmitted via pouches pedicles. They were interpreted as a colicky lower abdominal pain (gas pain) and not as a need to void. However, by time and training, it was translated as a symptom of urine distension.

Encouraging results of tumor downstaging by cisplatin-based 3/4 courses and further regimens for muscle invasive tumors, in addition to the inclusion of the high-risk T_1_ lesions that are located away from the bladder neck, enabled safe PRC. Both groups of patients represented over 70% of our total included patients. Long-term serial follow-up urethroscopy and cystoscopy found neither urethral nor pouch recurrence, and the entire delayed neck strictures that developed were benign fibrosis [4, 5, 14, 15, 19, 20].

Proximal urethral length preservation together with bladder neck sphincter improved significantly continence both subjective and in UDS. PRC voiding control was significantly better than RC starting with the 2nd month to the 5th year and this superiority was translated to each of the pouch configurations. Bladder neck and voluntary urethral pressures for the PRC group were above pouch pressure at the start and augmented by time leading in addition to nocturnal continence progress, marked decrease of the stress leaks.

### Emptying characteristics of the 3 neobladders

Use of the ileum alone to construct a bladder substitute remains associated with substantial advantages of distensibility and compliance. W-pouches have a large capacity from the beginning that assumes continence almost from the early postoperative period, in contrast to all other types of reservoirs, such as Y and the earlier Studer’s reservoirs, for which the early capacity is smaller and requires months until it reaches the ultimate volume and incontinence disappears [6–9, 11, 21, 22]. Basal detrusor pressure in our cases kept low for the three pouches along the study period, and maximal pressure increased over time. This explains the high volume capacity of 500 ml equal for the three pouches from the start, and the increased voiding volume over time.

Pad-free W-daytime continence was perfect in half of our series starting from the early postoperative period, though 85% had accompanying infrequent stress incontinence. Only 13% of patients had pad-free nighttime dryness. By the 6th month, 65% of the cases with PRC were continent by day and 45% by night, with slightly lower rates for RC. At the end of the 5th year, 68% were 24-h overall continent and 90 and 70% day and night. Hautmann and others, after 121 months median follow up, observed a good daytime and nighttime continence around 90% and 82%, respectively, and 87% void spontaneously with less than 100 ml of residual urine. However, the overall pad-free rate was markedly lower at 71%/47%, respectively.^7^

By the 6th month, W, Y, and IC cases with PRC had equivalent control rates because the three pouches reached equivalent capacities with higher residual volume in the W group and upper maximal reservoir pressure in the IC group.

IC series had an average 11-month to achieve night-time control equal to that for ileal pouches [6–15, 17, 21–23]. Nevertheless, continence improved with time in a good percentage of cases by pelvic floor training and decreasing fluid intake before bedtime.

Night control reports for variable ileal neobladders also required 6 to12 months to reach maximum levels as the capacity and compliance increase; nevertheless, nighttime leakage was 20–30% [7, 17, 21–23].

This delay is partly due to the nocturnal output of urine which exceeded the reservoirs’ capacities because of the shifted water by the intestine to render the concentrated nocturnal urine iso-osmolar. This shift decreases with time together with adaptation refining continence with time. Moreover, loss of the afferent limb of the sacral bladder-sphincter reflex in RC candidates prevents the increase in sphincter tone in response to the increase in pouch volume resulting in relative sphincter insufficiency. This is not the case for PRC candidates with intact reflex. These factors together with weakness of the altered feedback to the brain stem via the neobladder afferents which awake the patient when the reservoir is full result in a relatively high frequency of nocturnal enuresis but it improves gradually with time when the new sensation of fullness appears and by evacuation at fixed times, at least once per night. Late nocturnal incontinence was less in general for the 3-pouches after PRC more noticeable for the IC. Nerve sparing together with a functional proximal prostatic urethra regains undisturbed spinal reflex which leads to an immediate increase in sphincter activity particularly the internal one during augmented intra-abdominal pressure. This describes why many of our patients monitored later than 3 years had no stress incontinence when coughing or straining. Also, the increased functional urethral length made it under intra-abdominal pressure.

Parameters of the outlet (maximal outlet pressure, functional length, and maximal outlet closure pressure) were the same for the various reservoirs. However, the reservoir parameters (functional capacity, cyst metric capacity and maximal contraction pressure) differ significantly at the early times depending on the segment used (ileum or colon) and the specific segment length used for its construction; however, by delayed surveillances, pouch parameters unified.

### Morbidity

Early neobladder related complications compare favorably with those reported by other studies, and most of them were avoidable and of simple curable CDC grades. Urine and intestinal leaks were 21%, 10%, and 12% for W, Y, and IC, respectively, and mostly CDC II. Unrelated complications were 7% for each of Y and IC alone.

### Delayed

Total delayed morbidities rates were greater for W series than either Y or IC, principally for the over-distension and electrolyte disturbances (Table 1).

Reflux prevention was a less important issue in ureteroenteric anastomosis because of the low maximal pouch pressure and the discovery that employment of any antireflux technique by itself would double the risk of strictures [9, 24]. Delayed refluxes developed in 7% of the total study group, and all of them had associated neck stricture and overdistension.

Recent meta-analysis detected higher incidence of ureteroenteric strictures in anti-reflux anastomosis and additionally refluxes with direct implantation were not even directly related to impairments or pyelonephritis [9]. Regarding our uretero-enteric strictures, Y-group were the most frequent of the three versions and commonly short. Long strictures more than 2 cm were limited and lend well to endo-ureterotomy ± open repair [25].

Renal units usually remain stable without back-pressure effects in 79% [1–3, 7, 9]. Likewise, in this ongoing study, 20/24 of renal units with preoperative hydroureters improved after 1 to 2 months with neither later on kidney’s parenchyma deteriorations nor ureteric tumor recurrence [26, 27].

Preserving the distal part of the terminal ileum keeps normal absorption of vita. B12, folic acid, and bile salts, decreasing postoperative diarrhea and B12 deficiency. In our study, early transient diarrhea was encountered in the 3 pouch groups and responded to constipating measures without delayed, long-lasting diarrhea [28].

Short periods of disturbed bicarbonate base defect more than − 2.5 mEq/L were frequent with W (40%) followed by Y (25%) and least observed in IC (12%). This observation is in support of IC configuration.

In our total study, patients 27% suffered mild temporary metabolic acidosis, and 9 cases developed persistent metabolic acidosis, predisposed by pouch problems. Metabolic disturbances were mostly limited to the first postoperative year and disappeared by improving nocturnal control, signifying that not only the absorptive ability is responsible for the acidosis, but the time of urine storage.

Reports using W, Y, IC, Mainz, and Studer ileal neobladder pouch showed that total patients demonstrated early metabolic acidosis and received sodium bicarbonate 2 to 6 g/day for 3 to 6 weeks [6, 8, 11, 17, 22, 27].

The higher ability to accommodate intraluminal pressure seems to be limitless in ileal pouches, particularly W. This factor, in addition to the delayed or lost neo-vesicle sensation, leads to overdistension and sometimes spontaneous rupture. Delayed ruptures have been reported on top of acute or chronic overdistention observed with long-term follow-up, as ileal pouch capacity enlarges and the efficiency of voiding by Valsalva’s straining reduces. Overdistension could partly develop because of delayed voiding due to pouch defective sense in part of the patient or neck obstruction. Atrophy of the smooth muscular layer of the ileal wall and fibrous tissues replacing it over time leads to the thin overdistended pouch walls and the large residual [29].

## Conclusions

Preserving prostatic urethra and bladder neck internal sphincter together with nerve sparing in selected bladder cancers greatly improved day and night continence devoid of urethral recurrence. This advantage is translated to whatsoever pouch configuration. Early continence is supporting W shaped ileal pouch more than Y-shaped or ileocecal pouches; but by the 6th month and afterwards they are equal. Incidence of pouch related complications in W patients is higher than both Y and IC. Spherical IC has a lower over distension rate (5%) in contrast to W group (30%) and Y cases (22.5%).

## Data Availability

The original series characteristic data within this article including figures have been deposited in the National Cancer Institute Archives  and available on reasonable authorized request from the corresponding author.

## Abbreviations

IC: Spherical ileocoecal pouch
RC: Radical cystoprostatectomy
PRC: Prostate sparing radical cystectomy
W: Ileal pouch in W shaped configuration
Y: Ileal pouch in Y shaped configuration
CDC: Clavien-Dindo complication grading system
UDS: Urodynamic study
EBRT: External beam radiation therapy
